# Supplementary material for: Nature and Mental Health in Urban Texas: A NatureScore-Based Study
Source: Int J Environ Res Public Health. 2024 Feb 1;21(2):168. doi: 10.3390/ijerph21020168 (PMC10887946; doi:10.3390/ijerph21020168)

Cross-Reference Intelligence Process to Correct Averaging in Remote Sensing Phenology.

Phenology studies use data gathered by satellite sensors that measure wavelengths of light absorbed and reflected by green plants. Certain pigments in plant leaves strongly absorb wavelengths of visible (red) light. Many sensors carried aboard satellites measure red and near-infrared light waves reflected by land surfaces. Using mathematical formulas (algorithms), scientists transform raw satellite data about these light waves into vegetation indices. A vegetation index is an indicator that describes the greenness — the relative density and health of vegetation — for each picture element, or pixel, in a satellite image. Although there are several vegetation indices, one of the most widely used is the Normalized Difference Vegetation Index (NDVI). NDVI values range from +1.0 to -1.0. Areas of barren rock, sand, or snow usually show very low NDVI values (for example, 0.1 or less). Sparse vegetation such as shrubs and grasslands or senescing crops may result in moderate NDVI values (approximately 0.2 to 0.5). High NDVI values (approximately 0.6 to 0.9) correspond to dense vegetation such as that found in temperate and tropical forests or crops at their peak growth stage. NDVI scores for water generally have negative values.

Depending on the quality of the satellite data, the pixel data corresponds to ~10-30 meters of surface area on the earth. To measure the volume or health of vegetation in a larger area, an average of the aggregated pixel data is typically used. One problem when attempting to determine the volume of natural versus built elements for a large area using NDVI averaging is that many natural elements (like water or rock or sand) can offset abundant adjacent live vegetation. For example, if you looked at a 100 sq. meter circle that was half jungle and half water, you would get a score that looks identical to a parking lot (since the negative water score and positive jungle average out to zero).

As seen in the sample images below, using a NDVI averages for the circle above in the lower right corner, you would get an indication that the area had equal to or less natural elements than the adjacent neighborhoods despite being predominantly park space and water.

Another similar issue is that various built elements, like different rooftop colors or types of concrete, may skew NDVI values erroneously.

NatureQuant has developed a cross-referencing system that combines NDVI averaging with other land classification datasets and remote sensing technologies to allow for an accurate measurement of the density/volume of live vegetation and the balance between built and natural elements. Using the NatureQuant process you can get a determination what percentage of an area is natural versus built elements and, among the natural elements, how much of it is live, health vegetation.

NatureQuant has also developed a process to correct various data sources for edge cases, or other scenarios where an element (e.g., water, artificial turf fields, agricultural land, colored roofs) is not correctly measured as “nature”, due to the technique used to capture the data. The following components are considered in that measure: computer vision of aerial/street Images; cloud- and water-corrected

normalized difference vegetation index (NDVI) based on an annual average, sampled every 16 days; normalized difference water index (NDWI) processed satellite imagery that measures water availability; water quality data (select areas); parks geographic information system (GIS) data; park features through examining the various park amenities (playgrounds, sport courts, etc.) and park quality ratings from public platforms (Yelp, Foursquare, Google Reviews, etc.); natural/nature features GIS data; GIS and land classification databases; impervious surface data that measures the amount of artificial structures (concrete, pavement, bricks, asphalt, etc.); road and building density data; human modification (HM) model for landscape integrity. For each location, NatureScore analyzes and blends various data sets and processed information.; air quality index (AQI); noise pollution; light pollution; ultraviolet exposure (watt-hours per square meter); tree canopy data; tree biomass and species identification (select areas); light detection and ranging (LIDAR) (select areas); tasseled cap values data structures; global biodiversity information facility (Order/Family/Genus/Species Data). A machine learning algorithm was used to combine the various variables for each zipcode included in the analysis and estimate a NatureScore for each one according to the available nature elements. The considered elements are weighted to create the highest correlation between geo-tagged longevity averages and the given natural elements via a machine learning process. This process is repeatedly updated as more health outcome and environmental data is collected. Note that certain "natural" elements that have not demonstrated positive health correlations, like sand or rock, are therefore less valuable than live vegetation. The NatureScore values range from zero (poor NatureScore, lacking beneficial natural elements) to 100 (high NatureScore, abundant beneficial natural elements). For this study, the NatureScore data was calculated via an examination of the elements within the provided zipcode polygons based on 2019 data, and it was categorized into four groups: Nature Deficient/Nature Light (0-39), Nature Adequate (40-59), Nature Rich (60-79), and Nature Utopia (80-100).

One of the limitations of NatureScore was that it is designed to allow for high location granularity (down to every 10 meters), which can provide a unique score for any home address. When examining a geographical area as large as zipcode, NatureScores will skew towards higher scores as more natural areas and park spaces are inevitably captured.

NatureScore™

By Nature Quant

National Land Classification Database

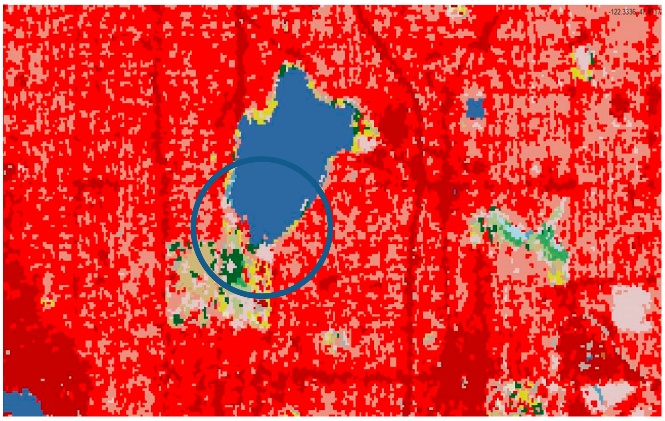

Open Street Maps / Park Maps

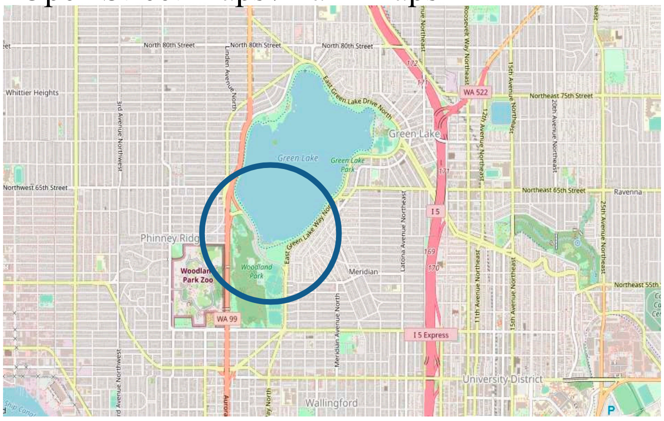

Computer Vision / Street & Satellite Imagery

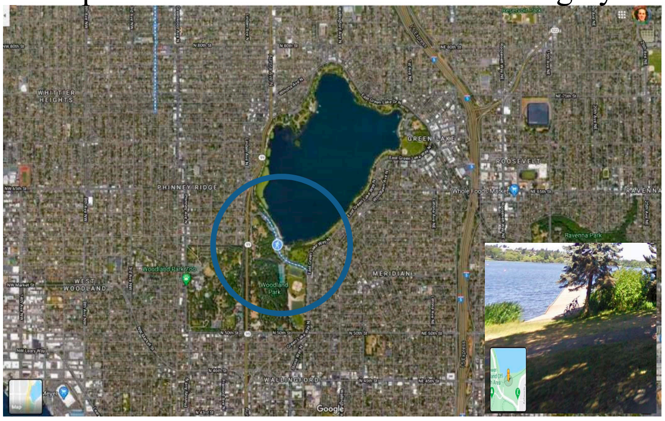

Normalized Difference Vegetation Index

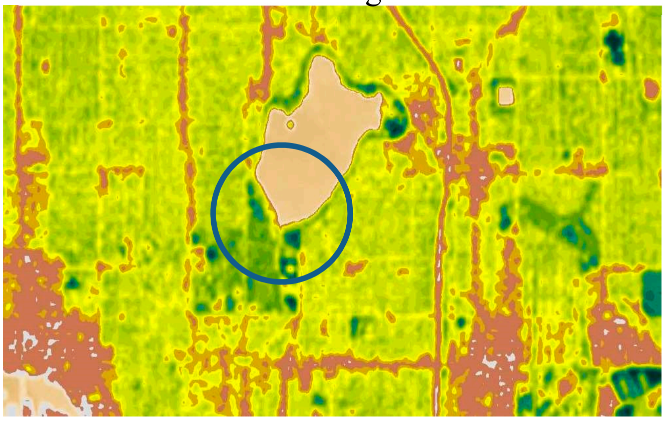

Supplement: Supplementary file 1 [file ijerph-21-00168-s001.zip › File S2. NatureScore.pdf]
