# Supplementary material for: Nature and Mental Health in Urban Texas: A NatureScore-Based Study
Source: Int J Environ Res Public Health. 2024 Feb 1;21(2):168. doi: 10.3390/ijerph21020168 (PMC10887946; doi:10.3390/ijerph21020168)
Supplement: Supplementary file 1 [file ijerph-21-00168-s001.zip › Supplementary Figures and Tables.pdf]

Supplementary Figures

Figure S1. Correlation matrix showing the relationship between the various socio-economic factors and NatureScore.

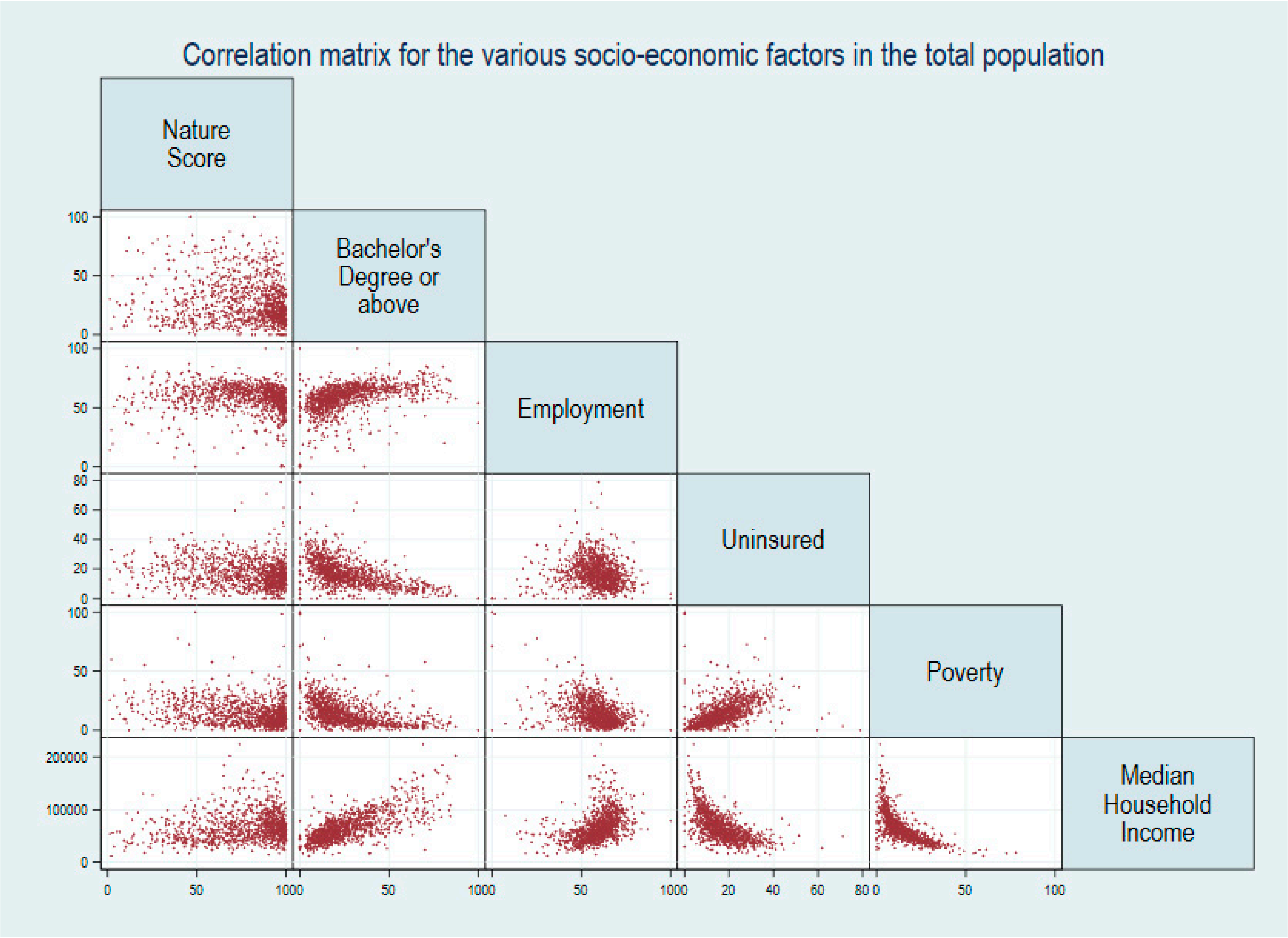

Figure S2. Satellite images of neighborhoods with different NatureScores

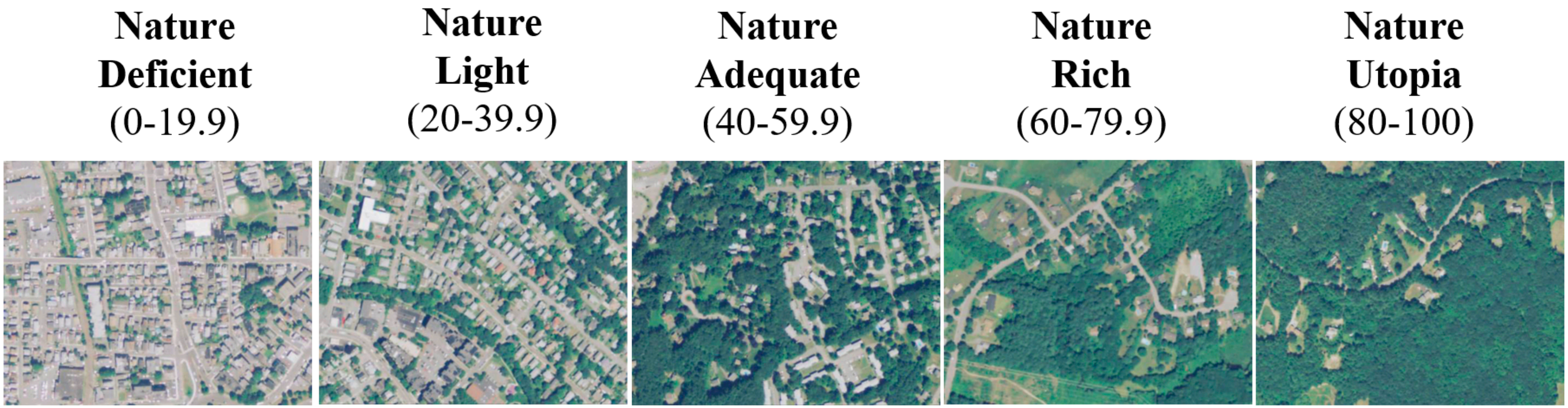

Supplementary Tables

Table S1. List of ICD-10 CM and ICD-9 codes used to define depression.

ICD-10 CM / ICD-9	DESCRIPTION
**F320**	Major depressive disorder, single episode, mild
**F321**	Major depressive disorder, single episode, moderate
**F322**	Major depressive disorder, single episode, severe without psychotic features
**F323**	Major depressive disorder, single episode, severe with psychotic features
**F324**	Major depressive disorder, single episode, in partial remission
**F325**	Major depressive disorder, single episode, in full remission
**F3289**	Other specified depressive episodes
**F329**	Major depressive disorder, single episode, unspecified
**F32A**	Depression, unspecified
**F330**	Major depressive disorder, recurrent, mild
**F331**	Major depressive disorder, recurrent, moderate
**F332**	Major depressive disorder, recurrent severe without psychotic features

**F333**	Major depressive disorder, recurrent, severe with psychotic symptoms
**F3340**	Major depressive disorder, recurrent, in remission, unspecified
**F3341**	Major depressive disorder, recurrent, in partial remission
**F3342**	Major depressive disorder, recurrent, in full remission
**F338**	Other recurrent depressive disorders
**F339**	Major depressive disorder, recurrent, unspecified
**29620**	Major depressive affective disorder, single episode, unspecified
**29621**	Major depressive affective disorder, single episode, mild
**29622**	Major depressive affective disorder, single episode, moderate
**29623**	Major depressive affective disorder, single episode, severe, without mention of psychotic behavior
**29624**	Major depressive affective disorder, single episode, severe, specified as with psychotic behavior
**29625**	Major depressive affective disorder, single episode, in partial or unspecified remission
**29626**	Major depressive affective disorder, single episode, in full remission
**29630**	Major depressive affective disorder, recurrent episode, unspecified
**29631**	Major depressive affective disorder, recurrent episode, mild
**29632**	Major depressive affective disorder, recurrent episode, moderate

**29633**	Major depressive affective disorder, recurrent episode, severe, without mention of psychotic behavior
**29634**	Major depressive affective disorder, recurrent episode, severe, specified as with psychotic behavior
**29635**	Major depressive affective disorder, recurrent episode, in partial or unspecified remission
**29636**	Major depressive affective disorder, recurrent episode, in full remission
**29682**	Atypical depressive disorder
**311**	Depressive disorder, not elsewhere classified

Table S2. List of ICD-10 CM and ICD-9 codes used to define bipolar disorder.

ICD-10 CM / ICD-9	DESCRIPTION
**F310**	Bipolar disorder, current episode hypomanic
**F3110**	Bipolar disorder, current episode manic without psychotic features, unspecified
**F3111**	Bipolar disorder, current episode manic without psychotic features, mild
**F3112**	Bipolar disorder, current episode manic without psychotic features, moderate
**F3113**	Bipolar disorder, current episode manic without psychotic features, severe
**F312**	Bipolar disorder, current episode manic severe with psychotic features
**F3130**	Bipolar disorder, current episode depressed, mild or moderate severity, unspecified
**F3131**	Bipolar disorder, current episode depressed, mild
**F3132**	Bipolar disorder, current episode depressed, moderate
**F314**	Bipolar disorder, current episode depressed, severe, without psychotic features
**F315**	Bipolar disorder, current episode depressed, severe, with psychotic features
**F3160**	Bipolar disorder, current episode mixed, unspecified
**F3161**	Bipolar disorder, current episode mixed, mild

**F3162**	Bipolar disorder, current episode mixed, moderate
**F3163**	Bipolar disorder, current episode mixed, severe, without psychotic features
**F3164**	Bipolar disorder, current episode mixed, severe, with psychotic features
**F3170**	Bipolar disorder, currently in remission, most recent episode unspecified
**F3171**	Bipolar disorder, in partial remission, most recent episode hypomanic
**F3172**	Bipolar disorder, in full remission, most recent episode hypomanic
**F3173**	Bipolar disorder, in partial remission, most recent episode manic
**F3174**	Bipolar disorder, in full remission, most recent episode manic
**F3175**	Bipolar disorder, in partial remission, most recent episode depressed
**F3176**	Bipolar disorder, in full remission, most recent episode depressed
**F3177**	Bipolar disorder, in partial remission, most recent episode mixed
**F3178**	Bipolar disorder, in full remission, most recent episode mixed
**F3181**	Bipolar II disorder
**F3189**	Other bipolar disorder
**F319**	Bipolar disorder, unspecified
**29600**	Bipolar I disorder, single manic episode, unspecified

**29601**	Bipolar I disorder, single manic episode, mild
**29602**	Bipolar I disorder, single manic episode, moderate
**29603**	Bipolar I disorder, single manic episode, severe, without mention of psychotic behavior
**29604**	Bipolar I disorder, single manic episode, severe, specified as with psychotic behavior
**29605**	Bipolar I disorder, single manic episode, in partial or unspecified remission
**29606**	Bipolar I disorder, single manic episode, in full remission
**29640**	Bipolar I disorder, most recent episode (or current) manic, unspecified
**29641**	Bipolar I disorder, most recent episode (or current) manic, mild
**29642**	Bipolar I disorder, most recent episode (or current) manic, moderate
**29643**	Bipolar I disorder, most recent episode (or current) manic, severe, without mention of psychotic behavior
**29644**	Bipolar I disorder, most recent episode (or current) manic, severe, specified as with psychotic behavior
**29645**	Bipolar I disorder, most recent episode (or current) manic, in partial or unspecified remission
**29646**	Bipolar I disorder, most recent episode (or current) manic, in full remission
**29650**	Bipolar I disorder, most recent episode (or current) depressed, unspecified
**29651**	Bipolar I disorder, most recent episode (or current) depressed, mild

**29652**	Bipolar I disorder, most recent episode (or current) depressed, moderate
**29653**	Bipolar I disorder, most recent episode (or current) depressed, severe, without mention of psychotic behavior
**29654**	Bipolar I disorder, most recent episode (or current) depressed, severe, specified as with psychotic behavior
**29655**	Bipolar I disorder, most recent episode (or current) depressed, in partial or unspecified remission
**29656**	Bipolar I disorder, most recent episode (or current) depressed, in full remission
**29660**	Bipolar I disorder, most recent episode (or current) mixed, unspecified
**29661**	Bipolar I disorder, most recent episode (or current) mixed, mild
**29662**	Bipolar I disorder, most recent episode (or current) mixed, moderate
**29663**	Bipolar I disorder, most recent episode (or current) mixed, severe, without mention of psychotic behavior
**29664**	Bipolar I disorder, most recent episode (or current) mixed, severe, specified as with psychotic behavior
**29665**	Bipolar I disorder, most recent episode (or current) mixed, in partial or unspecified remission
**29666**	Bipolar I disorder, most recent episode (or current) mixed, in full remission
**2967**	Bipolar I disorder, most recent episode (or current) unspecified

**29680**	Bipolar disorder, unspecified
**29681**	Atypical manic disorder
**29689**	Other bipolar disorders

Table S3. List of ICD-10 CM and ICD-9 codes used to define anxiety disorders.

ICD-10 CM / ICD-9	DESCRIPTION
**F410**	Panic disorder [episodic paroxysmal anxiety]
**F411**	Generalized anxiety disorder
**F412**	Mixed anxiety and depressive disorder
**F413**	Other mixed anxiety disorders
**F418**	Other specified anxiety disorders
**F419**	Anxiety disorder, unspecified
**30000**	Anxiety state, unspecified
**30001**	Panic disorder without agoraphobia
**30002**	Generalized anxiety disorder
**30009**	Other anxiety states

Table S4. List of ICD-10 CM and ICD-9 codes used to define stress disorders.

ICD-10 CM / ICD-9	DESCRIPTION
**F430**	Acute stress reaction
**F4310**	Post-traumatic stress disorder, unspecified
**F4311**	Post-traumatic stress disorder, acute
**F4312**	Post-traumatic stress disorder, chronic
**F4320**	Adjustment disorder, unspecified
**F4321**	Adjustment disorder with depressed mood
**F4322**	Adjustment disorder with anxiety
**F4323**	Adjustment disorder with mixed anxiety and depressed mood
**F4324**	Adjustment disorder with disturbance of conduct
**F4325**	Adjustment disorder with mixed disturbance of emotions and conduct
**F4329**	Adjustment disorder with other symptoms
**F4381**	Prolonged grief disorder
**F4389**	Other reactions to severe stress

**F439**	Reaction to severe stress, unspecified
**3090**	Adjustment disorder with depressed mood
**3091**	Prolonged depressive reaction
**30921**	Separation anxiety disorder
**30922**	Emancipation disorder of adolescence and early adult life
**30924**	Adjustment disorder with anxiety
**30928**	Adjustment disorder with mixed anxiety and depressed mood
**30929**	Other adjustment reactions with predominant disturbance of other emotions
**3093**	Adjustment disorder with disturbance of conduct
**3094**	Adjustment disorder with mixed disturbance of emotions and conduct
**30981**	Posttraumatic stress disorder
**30982**	Adjustment reaction with physical symptoms
**30983**	Adjustment reaction with withdrawal
**30989**	Other specified adjustment reactions
**3099**	Unspecified adjustment reaction
**3083**	Other acute reactions to stress

**3084**	Mixed disorders as reaction to stress
**3089**	Unspecified acute reaction to stress

Table S5. Distribution of Neighborhood NatureScore groups

NatureScore group	N (%)	Mean (SD)	Median (IQR)	Range (Min-Max)
**Overall**	1,169 (100)	77.21 (21.96)	85.8 (64.8–94.7)	1.2 – 100
**Nature Deficient / Nature Light (0-39)**	94 (8.04)	26.65 (10.94)	30.35 (19.5–36.8)	1.2 – 39.9
**Nature Adequate (40-59)**	159 (13.60)	51.07 (6.01)	50.3 (46.7–57.0)	40 – 59.9
**Nature Rich (60-79)**	246 (21.04)	71.03 (5.57)	71.5 (66.6–75.9)	60.3 – 79.7
**Nature Utopia (80-100)**	670 (57.31)	92.76 (5.25)	93.4 (89.1–97.4)	80 – 100

N: Number of zipcodes; %: percentage; SD: standard deviation; IQR: inter-quartile range; Min: minimum; Max: maximum

Table S6. Multivariable regression results for depression stratified by black population in each zipcode.

	Black population <10%		Black population 10+%	
**No. of Zipcodes**	700		416	
	**aRR (95%CI)**	**P-value**	**aRR (95%CI)**	**P-value**
**NatureScore Categories**				
**Nature Deficient / Nature Light**	Reference		Reference	
**Nature Adequate**	1.221 (0.913 - 1.632)	0.178	0.905 (0.529 - 1.548)	0.716
**Nature Rich**	1.103 (0.866 - 1.404)	0.427	0.737 (0.432 - 1.259)	0.265
**Nature Utopia**	0.930 (0.751 - 1.152)	0.509	0. 724 (0.419 – 1.253)	0.249

aRR: adjusted rate ratio; CI: confidence interval; No: number; %: percentage

Table S7. Multivariable regression results for bipolar stratified by black population in each zipcode.

**Stratification by Hispanic Population in Zipcode**				
	**Hispanic population <10%**		**Hispanic population 10+%**	
**No. of Zipcodes**	594		422	
	**aRR (95%CI)**	**P-value**	**aRR (95%CI)**	**P-value**
**NatureScore Categories**				
**Nature Deficient / Nature Light**	Reference		Reference	
**Nature Adequate**	1.074 (0.566 – 2.039)	0.826	0.689 (0.448 - 1.062)	0.092
**Nature Rich**	1.090 (0.572 – 2.078)	0.793	0.719 (0.475 – 1.091)	0.121
**Nature Utopia**	0.744 (0.392 - 1.412)	0.366	0.629 (0.404 – 0.979)	**0.040**
**Stratification by Educational Attainment in Zipcode**				
	**Educational Attainment <25%**		**Educational Attainment 25+%**	
**No. of Zipcodes**	561		455	
	**aRR (95%CI)**	**P-value**	**aRR (95%CI)**	**P-value**
**NatureScore Categories**				
**Nature Deficient / Nature Light**	Reference		Reference	

**Nature Adequate**	1.398 (1.019 – 1.916)	**0.037**	0.425 (0.214 – 0.844)	**0.014**
**Nature Rich**	1.363 (1.011 – 1.837)	**0.042**	0.391 (0.197 – 0.772)	**0.007**
**Nature Utopia**	1.264 (0.952 - 1.679)	0.106	0.311 (0.159 – 0.608)	**0.001**

aRR: adjusted rate ratio; CI: confidence interval; No: number; %: percentage
